# Evaluation of the efficacy of auricular acupressure in preventing depression in adolescents with insomnia: study protocol for a randomized controlled trial

**DOI:** 10.3389/fpsyt.2025.1613052

**Published:** 2025-09-04

**Authors:** Xiaoyang Lian, Yuting Zhang, Min Xu, Xiaojie Li, Guoyu Li, Xiaoying Wang, Shan Qin, Junlan Ye, Chengyong Liu

**Affiliations:** ^1^ Affiliated Hospital of Nanjing University of Chinese Medicine, Jiangsu Province Hospital of Chinese Medicine, Nanjing, Jiangsu, China; ^2^ Yueyang Hospital of Integrated Traditional Chinese and Western Medicine, Shanghai University of Traditional Chinese Medicine, Shanghai, China; ^3^ College of Integrative Chinese and Western Medicine, Jiangsu Health Vocational College, Nanjing, Jiangsu, China; ^4^ Chongqing Hospital of the First Affiliated Hospital of Guangzhou University of Traditional Chinese Medicine, Chongqing, China; ^5^ Affiliated Hospital of Nanjing University of Chinese Medicine, Liyang Branch of Jiangsu Province Hospital of Chinese Medicine, Liyang, Jiangsu, China

**Keywords:** adolescent depression, adolescent insomnia, auricular acupressure, randomized clinical trial, study protocol

## Abstract

**Background:**

Insomnia and depression represent prevalent issues during adolescence. Auricular acupressure (AA), a simple, low-cost traditional Chinese medicine therapy with minimal adverse effects, has demonstrated efficacy in improving sleep quality and alleviating depressive symptoms in adult populations. However, existing research has predominantly focused on adults, with limited evidence regarding AA’s effectiveness in improving sleep and preventing depression among adolescents.

**Methods:**

This study is a single-center, double-blind, randomized controlled trial conducted in Nanjing, China. Participants are students from junior and senior high schools in Nanjing. A multi-stage stratified cluster sampling method is used to recruit students from 7th and 8th grades (junior high) and 10th and 11th grades (senior high). Baseline data will be collected using the Chinese version of the Pittsburgh Sleep Quality Index (PSQI), Beck Depression Inventory (BDI), Adolescent Sleep Hygiene Scale (ASHS), Children’s Depression Inventory (CDI), Adolescent Self-rating Life Events Checklist (ASLEC) and Adolescent Sleep-Wake Scale (ASWS), and insomnia patients will be identified. Adolescents who met the inclusion and exclusion criteria and whose guardians signed informed consent forms will be enrolled in the study. They will be randomly assigned to one of three groups: AA group, sham auricular acupressure (SAA) group, and control group. The treatment lasted for 8 weeks, with follow-up assessments at 3 and 6 months post-treatment. The primary outcome measures are PSQI and BDI scores, while secondary outcome measures included ASHS, CDI, ASLEC, and ASWS scores.

**Discussion:**

This prospective study evaluates AA’s efficacy in enhancing sleep quality and preventing depression in adolescents. The findings will elucidate AA’s potential clinical applications for adolescent insomnia treatment and depression prevention, thereby providing evidence to support its broader implementation in this population.

**Clinical trial registration:**

http://itmctr.ccebtcm.org.cn/, identifier ITMCTR2024000343.

## 1 Introduction

Depression is one of the most common mental disorders, characterized primarily by persistent and significant low mood. Adolescence is a critical period for the onset of depression. Research shows that the incidence of depression rises sharply after puberty, with the prevalence of depression in children being less than 1%, while in adolescence it increases to more than 4%–5% (1). Recent studies indicate that the prevalence of mild to moderate depression among adolescents is as high as 20% (2). Most cases remain undiagnosed or untreated, thereby increasing the risk of suicide in adolescents (3).

Insomnia is closely related to depression, and in both the DSM-IV and the latest DSM-V diagnostic criteria (4, 5), insomnia symptoms are listed as core symptoms of depression. Furthermore, many symptoms of daytime functional impairment in the diagnosis of insomnia overlap with the clinical manifestations of depression, such as lack of energy or fatigue, mood swings, and somatic symptoms. This overlap in diagnostic criteria further suggests that insomnia and depression share significant clinical similarities. Increasing evidence indicates that insomnia is not only a symptom of depression but may also be a potential predictive factor for depression (6).

Adolescence represents a critical developmental period characterized by significant alterations in sleep behavior. During this phase, adolescents experience physiological changes including delayed circadian rhythms and diminished homeostatic sleep regulation (7), culminating in a “perfect storm” of sleep disturbances manifested as delayed sleep onset, insufficient sleep duration, and difficulties in sleep maintenance (8). At the physiological level, the delayed maturation of the suprachiasmatic nucleus results in circadian phase delay, coupled with impaired homeostatic regulation of sleep need, leading to prolonged wakefulness in adolescents (8). Concurrently, psychosocial factors such as academic stress, social demands, and blue light exposure from electronic devices further disrupt sleep-wake cycles (9). Epidemiological studies have confirmed that sleep problems in adolescents significantly increase depression risk (10, 11), with this association being validated by a prospective cohort study in Guangzhou, China, demonstrating a dose-response relationship between reduced sleep duration (AOR=0.86, 95%CI: 0.80–0.92) and poorer sleep quality with subsequent depressive symptoms (12). Notably, while a bidirectional relationship exists between insomnia and depression (13), longitudinal studies more frequently indicate that insomnia precedes depressive onset, with some cases traceable to the perinatal period (14). Neurobiological research further reveals that decreased N3 sleep and diminished delta power during adolescent brain reorganization (15) may impair functional connectivity in emotion-regulation neural circuits (e.g., prefrontal-amygdala pathways), thereby establishing the neurobiological basis for insomnia-depression progression. Collectively, this evidence underscores the crucial need to develop adolescent-specific insomnia interventions for depression prevention.

Auricular acupressure (AA) is a non-pharmacological therapy originating from China, widely used for treating insomnia and depression. With historical roots tracing back to the Huangdi Neijing and systematically developed into modern auricular therapy by French physician Paul Nogier in the 1950s (16), AA primarily employs magnetic beads or Vaccaria seeds to stimulate specific auricular points for physiological regulation. The auricle represents the only somatic region with direct vagus nerve distribution, providing a neuroanatomical foundation for AA’s mechanism of action. By stimulating auricular vagal branches, AA transmits neural signals to brain regions critically involved in depression and insomnia, including the solitary nucleus, dorsal raphe nucleus, hippocampus, and prefrontal cortex (17–19). This intervention not only enhances cerebral hemodynamics and increases regional blood flow (20) but also modulates the secretion of sleep-related hormones and neurotransmitters such as serotonin, γ-aminobutyric acid, and melatonin (21), thereby improving sleep quality. Clinical studies demonstrate AA’s efficacy in enhancing sleep efficiency, reducing sleep latency, prolonging total sleep time, and alleviating comorbid anxiety and depressive symptoms (22).

Notably, while AA has shown robust therapeutic effects in adult populations, high-quality studies focusing on adolescents remain scarce, particularly regarding prospective prevention of adolescent depression. Furthermore, conventional first-line treatments like cognitive behavioral therapy face implementation challenges in school settings due to time constraints and operational complexity. In contrast, AA offers distinct advantages including technical simplicity, minimal adverse effects (23), and suitability for group administration, making it particularly amenable for school-based interventions. These characteristics warrant further systematic investigation of AA’s potential in adolescent mental health management.

Based on this, we have designed a randomized controlled study to screen adolescent insomnia patients using a multi-stage stratified cluster sampling method and randomly assign them to three groups: AA group, sham auricular acupressur (SAA) group, and control group. The intervention lasts for 8 weeks, and follow-up will be conducted at 3 months and 6 months post-treatment to evaluate the therapeutic effects of AA on adolescent insomnia and its potential to prevent depression. This study aims to explore a simple, safe, and effective intervention method, providing scientific evidence for the prevention of adolescent depression.

## 2 Materials and methods

### 2.1 Study design

**Figure 1:**
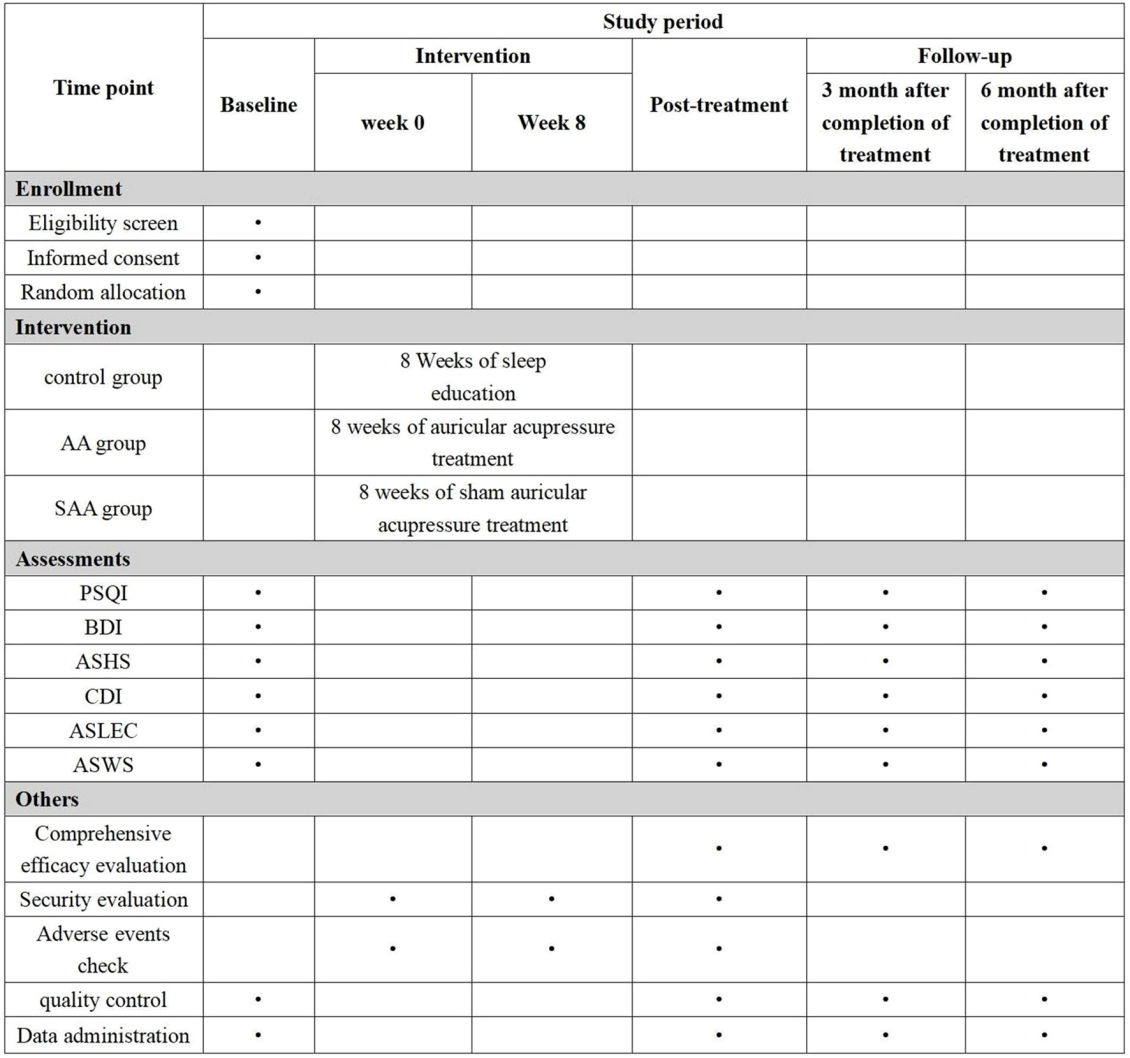
Flow diagram of the trial.

**Figure 2:**
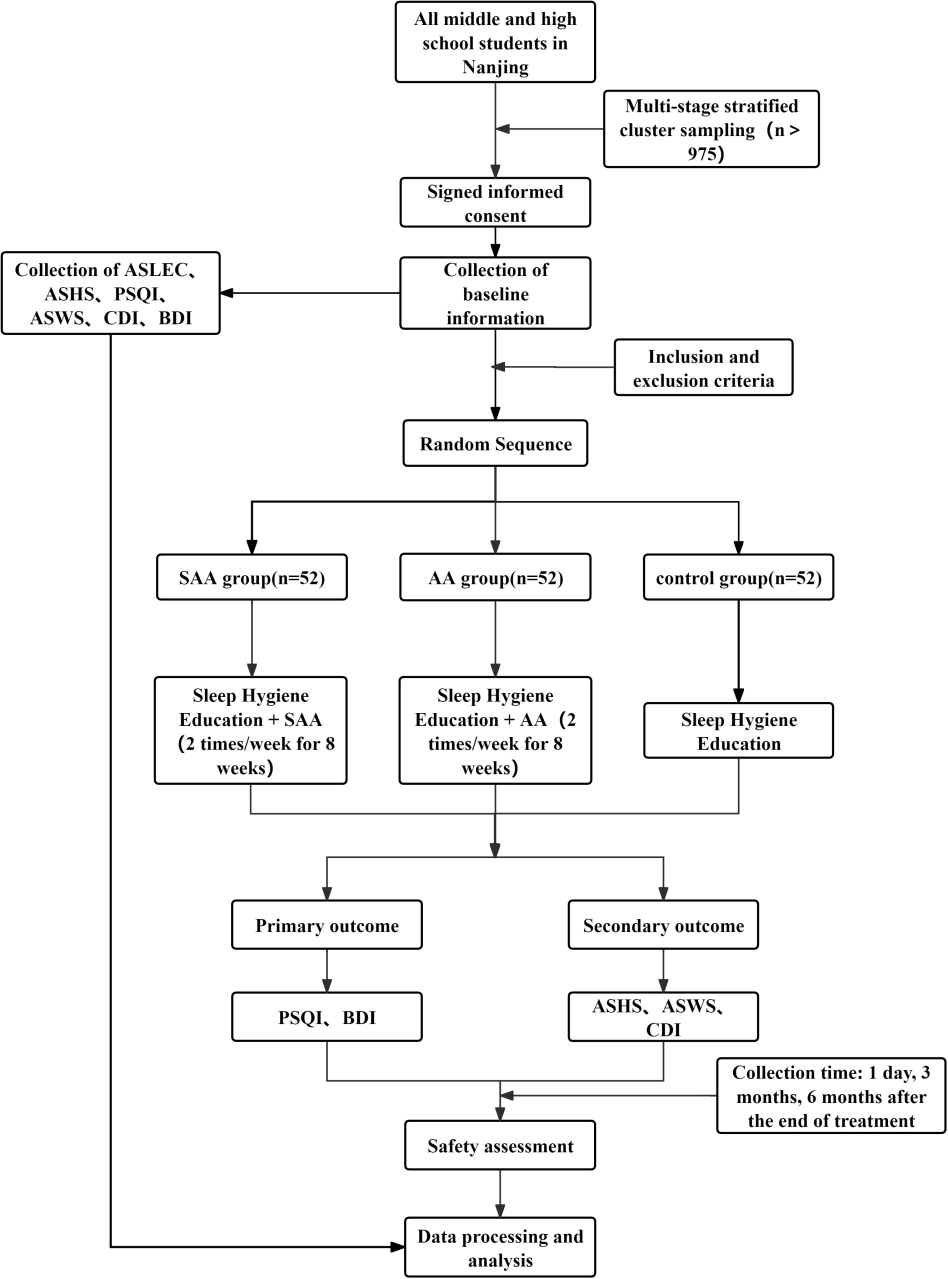
Schedule of enrolment, interventions, and assessments.

This study is a randomized controlled trial aimed at observing the efficacy of AA in treating insomnia and preventing depression in adolescents. The study protocol adheres to the SPIRIT guidelines. The study is conducted in Nanjing, and the participants are students from junior and senior high schools in Nanjing. A multi-stage stratified cluster sampling method is used to select students from the first and second grades of junior high school, and the first and second grades of senior high school. Adolescents with insomnia are identified and randomly assigned to three groups: AA group, SAA group, and control group. Adolescents with insomnia are diagnosed according to the third edition of the International Classification of Sleep Disorders (ICSD-3). Students who meet the inclusion criteria, along with their legal guardians, will be provided with an informed consent form. Students who agree to participate will be included in the study. The study flowchart is shown in **Figure 1**, and the timeline is shown in **Figure 2**.

### 2.2 Participants

This study is conducted in Nanjing, and the participants are students from junior and senior high schools in Nanjing. The study employed a multi-stage stratified cluster sampling approach, with the sampling process divided into three stages: educational level stratification, school stratification, and class sampling. First, schools were stratified by educational level into junior high school groups (grades 7 and 8) and senior high school groups (grades 10 and 11), excluding graduation classes (grades 9 and 12) to avoid potential interference from academic transitions on follow-up studies. Second, during school sampling, a stratified random sampling approach was adopted where junior and senior high schools were further substratified by geographic location (district, street/town) and school size (categorized into <800 students and ≥800 students based on enrollment numbers), ensuring proportional representation of each stratum relative to the overall population. Subsequently, four schools were independently and randomly selected from each stratum within both junior and senior high school groups. Finally, during class sampling, one target grade was randomly selected from grades 7 or 8 in junior high schools and grades 10 or 11 in senior high schools across the four sampled schools. For combined junior-senior high schools, their junior and senior sections were separately categorized into corresponding groups for independent sampling participation. In cases where a school had only one class in the target grade, that class was automatically included with documentation of this circumstance. Throughout the sampling process, detailed records of each school’s stratification attributes were maintained, and randomness in class selection was rigorously ensured.

#### 2.2.1 Inclusion criteria

(1) The diagnosis met ICSD-3 criteria, characterized by difficulty initiating sleep (>20 minutes), difficulty maintaining sleep, or early morning awakening despite adequate sleep opportunity, accompanied by daytime functional impairment. These symptoms occurred at least three times per week and persisted for more than three months.
(2) Age between 13–18 years;
(3) PSQI score > 7;
(4) No psychiatric medication treatment;
(5) No communication or cognitive impairments;
(6) Signed informed consent.

#### 2.2.2 Exclusion criteria

(1) Sleep disorders caused by organic diseases such as epilepsy, stroke, etc.;
(2) Sleep disorders caused by schizophrenia or other severe psychiatric disorders;
(3) Diagnoses of other sleep disorders, such as obstructive sleep apnea, REM sleep behavior disorder, or restless legs syndrome;
(4) Received AA therapy for insomnia in the past month;
(5) Alcohol and/or other drug abuse or dependence.

#### 2.2.3 Handling of dropouts

(1) Criteria for dropout

Patients who have provided informed consent, been screened and randomized for the trial, but for any reason do not complete the treatment and observation period as outlined in the study protocol will be considered dropouts.

(2) Handling of dropout cases

1) After a patient drops out, the researcher should contact the patient via home visits, scheduled follow-ups, phone calls, or letters to inquire about the reason for dropout, record recent sleep patterns, and complete any assessments that can be performed.

2) For patients who drop out due to allergies, adverse reactions, or treatment ineffectiveness, appropriate treatment measures should be taken according to their condition.

3) All dropout cases should be properly documented, both for archival purposes and for the required analysis in statistical evaluation. No additional patients will be recruited to replace dropouts.

#### 2.2.4 Trial termination

(1) If a serious adverse event occurs, or the researcher determines the trial should be stopped for this reason.
(2) If the patient’s condition worsens, or if other issues arise during the trial that affect observation, the researcher may decide to stop the trial.
(3) If significant protocol deviations occur during the implementation of the clinical trial, such as poor adherence to the protocol, making efficacy evaluation difficult, the trial may be stopped.
(4) If the participant expresses unwillingness to continue with the trial and requests to withdraw.

### 2.3 Sample size

Based on the type of study, the sample size is calculated using PASS 15.0 software. The PSQI score of insomnia patients is chosen as the outcome measure. Based on literature and preliminary experimental results, the average PSQI score for the drug control group is 10.76 ± 3.32, and it is expected that the PSQI score in the insomnia group will decrease by 2.63 points. An effect size of 0.8 is used, with α = 0.05 and power = 0.9. The calculated sample size for each group (AA group, SAA group, control group) is N1 = N2 = N3 = 52. Considering a 20% dropout rate, the total sample size required is N1 = N2 = N3 = 65. According to previous reports [5], the prevalence of insomnia in adolescents is 20%, so the sample population is (N1 + N2 + N3) ÷ 20% = 975, then the sample population should be more than 975.

### 2.4 Ethics and trial registration

The ethical review and informed consent materials for this study are approved by the Ethics Committee of the Nanjing University of Chinese Medicine Affiliated Hospital (Jiangsu Provincial Hospital of Chinese Medicine) on February 5, 2024. The approval number is 2024NL-010-02. This study has also been registered with the International Traditional Medicine Clinical Trial Registry, approval number ITMCTR2024000343. All invited participants and their legal guardians will receive a written informed consent document explaining the goals of the study and the participants’ rights. Participation is entirely voluntary.

### 2.5 Randomization

After screening based on the inclusion and exclusion criteria, eligible adolescent insomnia patients will be randomly assigned in a 1:1:1 ratio to the AA group, SAA group, or control group. The randomization sequence will be generated by researchers who are not involved in the trial implementation or statistical analysis using SPSS 25.0 software. The sequence will be hidden using the envelope method, and each envelope will contain the randomization order for patient allocation. A designated person will be responsible for patient inclusion, and patients will receive the corresponding envelope in the order of enrollment.

### 2.6 Blinding

To further improve the effectiveness of the blind method, participants in both the AA group and the SAA group were uniformly informed that they needed to apply pressure after applying the skin-colored tape, thereby reducing patients’ sensitivity to group differences. Additionally, the skin-colored tape used in both the AA and SAA groups was identical in appearance and texture, and was fixed by researchers at the same ear acupoint locations, ensuring that they could not be distinguished visually. Participants, evaluators, and statisticians will remain blinded throughout the study to minimize potential bias arising from procedural differences. As acupuncturists are responsible for delivering the AA and SAA interventions, they cannot be blinded to group allocation; however, they will not participate in outcome assessments. Evaluators will remain fully blinded to group assignments. They will solely be responsible for data collection and are prohibited from discussing treatment-related information with participants.

### 2.7 Interventions

#### 2.7.1 Control group

Sleep hygiene education: Actively communicate with the patient to understand the causes of insomnia, provide targeted guidance and explanations to alleviate negative emotions, and provide a sleep education manual. This includes informing patients about the importance of sleep, guiding them to create a good sleep environment, changing poor sleep habits, and engaging in regular physical exercise. The recommended exercise regimen consists of running (moderate-intensity aerobic activity) with a frequency of three sessions per week (24). Follow-up will be conducted regularly using ASHS, PSQI, ASWS, CDI, and BDI scales to assess sleep and psychological health. If the patient shows signs of depression, appropriate treatment will be provided.

#### 2.7.2 AA group

**Figure 3:**
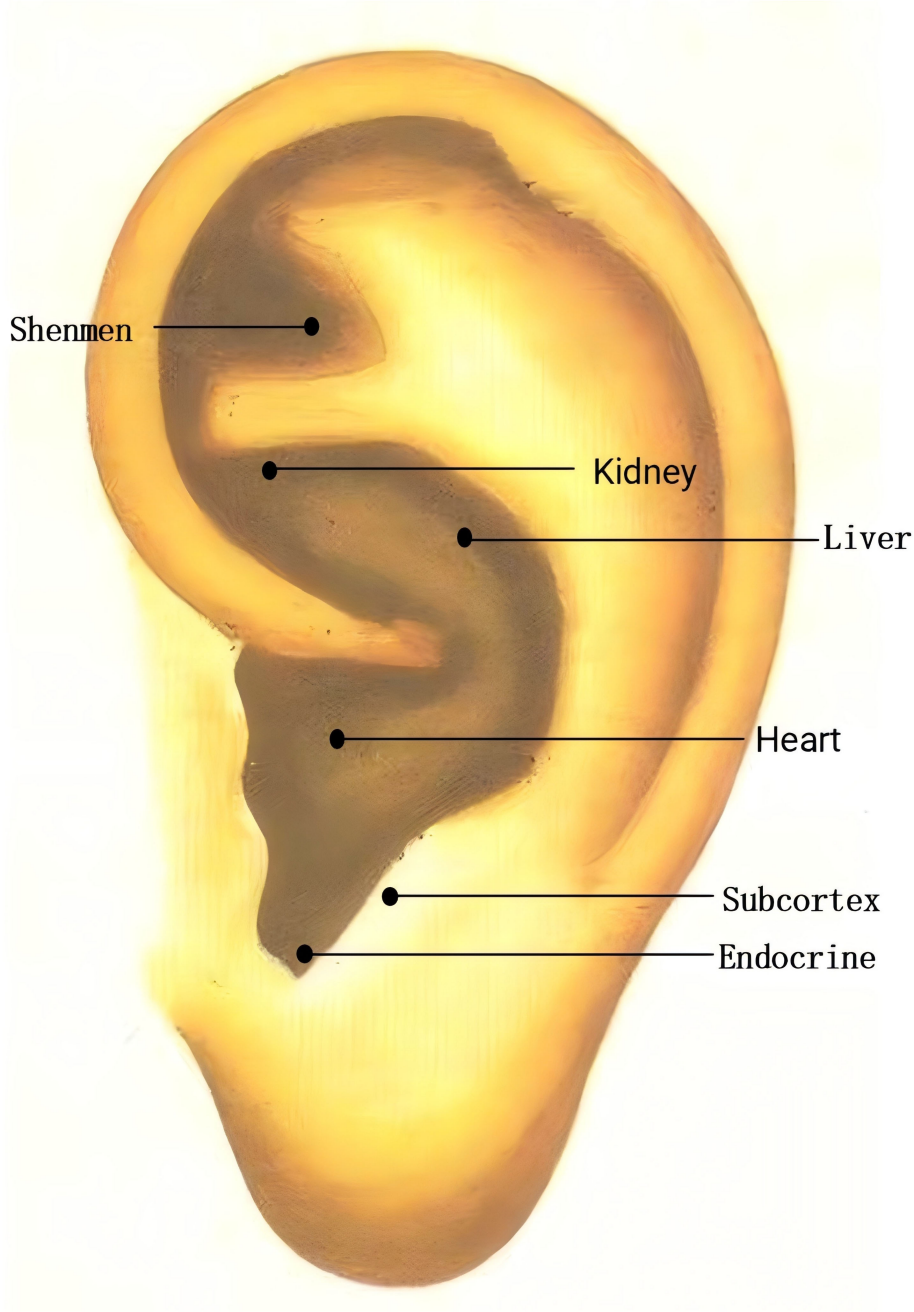
The accurate positioning of auricular points.

In the AA group, AA will be applied in addition to sleep hygiene education. Points: Shenmen (TF4, located in the triangular fossa, above the division of the ear lobe), Subcortex (AT4, on the inner side of the ear), Endocrine (CO18, anterior lower part of the ear canal), Liver (CO12, behind the lower part of the ear), Heart (CO15, central part of the ear), Kidney (CO10, below the upper and lower branches of the ear lobe) (**Figure 3**). These points will be carefully located according to the guidelines in the “Illustrated Ear Acupuncture” by Shanghai Science and Technology Publishing House. After evaluation, the most sensitive areas will be treated with ear acupressure beans. Each session will treat one ear, with changes to the other ear every 3 days. Patients will be instructed to press the acupressure beans 3–5 times per day, 1–2 minutes per session. The treatment will last for 8 weeks, followed by follow-ups at 3 months and 6 months.

#### 2.7.3 SAA group

In the SAA group, SAA will be applied in addition to sleep hygiene education. The same points as in the AA group will be selected, but adhesive tapes without seeds will be placed on the ear. To reduce intergroup differences and improve the effectiveness of the blinded method, the SAA group will also be required to press the ear patches according to uniform instructions to maintain consistency with the AA group’s operating procedures. This will prevent patients from easily guessing the groupings and ensure similarity in the operating experience between the two groups during the intervention. At the end of the study, patients will receive the same AA treatment as the ear acupressure group.

In addition, to minimize dropout rates and enhance participant adherence, researchers will engage with participants during each replacement of the ear acupressure beans. Participants will be reminded to apply pressure according to the prescribed frequency and duration, and to regularly monitor the placement and condition of the ear acupressure beans. These measures aim to encourage continued compliance with the study protocol.

### 2.8 Outcome assessment

#### 2.8.1 Primary outcome

PSQI: The Chinese version of the PSQI will be used for assessment (25, 26), which has been proven to be highly reliable and effective in evaluating the sleep quality of Chinese adolescents (27).The PSQI covers seven key dimensions: sleep quality, sleep latency, sleep duration, sleep efficiency, sleep disturbances, use of sleep medication, and daytime dysfunction. The total score ranges from 0 to 21, with higher scores indicating poorer sleep quality. A PSQI score > 7 is used as the cutoff for sleep quality problems.

BDI: A 21-item scale designed to assess core depressive symptoms, evaluating clinical manifestations over the past two weeks to determine depression severity, with total scores ranging from 0 to 63. For non-clinical adolescent populations, a BDI score ≥20 indicates the presence of depressive symptoms (28, 29).

#### 2.8.2 Secondary outcome

ASHS: A self-report measure assessing adolescents’ pre-sleep behavioral habits over the past month, including typical sleep routines and conditions, demonstrating robust psychometric properties (30). The scale consists of 28 items divided into 9 categories, with higher scores indicating better sleep hygiene. Categories include: Physiological domain (e.g., “I feel hungry when I go to bed”), Cognitive domain (e.g., “I go to bed thinking about things I need to do”), Emotional domain (e.g., “I feel anxious when I go to bed”), Sleep environment (e.g., “I fall asleep while watching TV”), Daytime sleep (e.g., “I nap for more than 1 hour during the day”), Substances (e.g., “I drink beer or other alcoholic beverages after 6 PM”), Bedtime (e.g., “I have a consistent bedtime”), Sleep stability (e.g., “I stay up later on weekends than my usual bedtime”), Bed/room sharing (e.g., “I sleep alone”)

CDI: CDI developed for children aged 6–16 years demonstrates good reliability and validity in Chinese pediatric populations (31), with participants selecting items that best describe their feelings over the past two weeks. The CDI consisted of 27 items divided into five dimensions: Anhedonia, Negative Mood, Negative Self-Esteem, Ineffectiveness, and Interpersonal Problem. Participants are asked to choose the item that best describes how they have felt in the past two weeks. Responses ranged from 0 to 2, indicating “often”, “occasionally”, and “always”, representing the frequency of occurrence, for a total score of 54. Lower scores indicate fewer depressive symptoms.

ASWS: The adolescent sleep quality self-report scale, clinically validated for reliability and validity (32), comprises 28 items designed to assess sleep quality in youth aged 12 to 18 years. Respondents are asked to rate the frequency of their sleep behaviors during the past month using a 6-point scale (ranging from “always” to “never”). Adolescent sleep is assessed across five behavioral dimensions: Bedtime (5 items), Sleep onset (6 items), Sleep maintenance (6 items), Sleep resumption (6 items), Waking up refreshed (5 items). An average subscale score for each dimension and a total sleep quality score (ASWS total score) can be obtained, with scores ranging from 1 to 6. Higher scores indicate better sleep quality.

ASLEC: A self-rating scale for negative life events specifically developed for adolescents by Chinese experts. The ASLEC assesses life events in the past six months at baseline, covering 27 items across six domains of negative life events in adolescents: interpersonal relationships, academic pressure, family conflicts, and health. The scale has sufficient test-retest reliability and concurrent validity, with total scores ranging from 0 to 108 (33).

### 2.9 Statistical analysis

Statistical analyses will be performed using SPSS 25.0 (SPSS, Chicago, IL, USA). The incidence of adolescent depression will be calculated based on the Beck Depression Inventory (BDI) thresholds established in prior studies. Continuous variables will be presented as mean ± standard deviation, while categorical data will be expressed as percentages or proportions. A p-value <0.05 will be considered statistically significant for intergroup differences, and p<0.01 will indicate highly significant differences.

#### 2.9.1 Intergroup comparison methods

For normally distributed measurement data meeting the assumptions of parametric tests, within-group pre- and post-treatment comparisons will be conducted using paired-sample t-tests. Intergroup comparisons will employ one-way ANOVA. Non-normally distributed measurement data will be analyzed using nonparametric rank-sum tests, and categorical data will be assessed via chi-square tests or Fisher’s exact probability test. These comparisons will evaluate the therapeutic effects of auricular acupressure.

#### 2.9.2 Multi-timepoint data analysis

For repeated-measures data satisfying the sphericity assumption, repeated-measures ANOVA will be applied. If the sphericity assumption is violated, Greenhouse-Geisser correction or multivariate ANOVA will be used. This analysis will examine the main effect of time on outcome measures and potential time-by-group interactions.

For non-normally distributed data or datasets with significant missing values across timepoints, mixed-effects models will be employed. These models accommodate unbalanced data and missing values while accounting for intra-individual correlations.

#### 2.9.3 Missing data handling and dropout analysis

Missing Data: Multiple imputation will be used to address missing data, leveraging intervariable correlations to generate plausible values and enhance result accuracy.

Dropout Analysis: The primary analysis will follow the per-protocol approach, including only subjects who adhered strictly to the study protocol. As a supplementary strategy, intention-to-treat (ITT) analysis will include all randomized participants regardless of protocol deviations or dropout status, preserving randomization integrity and minimizing selection bias. Additionally, baseline characteristics (e.g., age, sex, initial depression scores) of dropouts versus completers will be compared using chi-square or t-tests to assess systematic bias. Significant differences will prompt covariate adjustment in regression models.

#### 2.9.4 Correlation analysis and regression modeling

Independent t-tests and chi-square tests will identify significant associations (p<0.05) between depression and demographic features, sleep patterns, or life events. Significant factors will be incorporated into binary logistic regression models to evaluate their relationship with adolescent depression.

### 2.10 Safety evaluation

All participants will undergo a safety evaluation before and after treatment. Adverse events (e.g., localized skin redness, swelling, itching) that occur during treatment will be recorded. In case of an adverse event, the cause will be determined, its correlation with AA treatment will be assessed, and symptomatic treatment will be provided. In addition, all participants will undergo professional depression assessment, and depressive symptoms will be recorded on the Case Report Form (CRF). If severe depression is detected, the trial will be terminated, and further treatment will be provided.

Safety will be evaluated according to the following adverse reaction grading system: Grade 1: Safe, no adverse reactions; Grade 2: Generally safe, mild adverse reactions that do not require treatment and allow continuation of therapy; Grade 3: Safety concerns, moderate adverse reactions, treatable but continuing therapy allowed; Grade 4: Trial termination due to adverse reactions.

### 2.11 Quality control

The acupressure beans and adhesive tapes used in the study were uniformly provided by the researchers to ensure standardized specifications. Prior to implementation, all physicians administering the interventions practiced accurate localization on a simulated ear model and were provided with an annotated acupoint diagram to guide proper technique.

The PSQI, BDI, ASHS, ASWS, CDI and ASLEC scales will be assessed for completion eligibility by a professionally qualified physician. Prior to the start of the study, researchers will undergo training on the trial protocol, undergo consistency checks on symptom quantification standards, and sign a researcher declaration. Researchers strictly adhered to informed consent procedures to ensure participants fully understood trial requirements and provided comprehensive cooperation. For consented participants, paper-based questionnaires were distributed, and parents/guardians were contacted to facilitate questionnaire completion and verification. All collected information was required to be authentic, complete, and verifiable against source documents.

### 2.12 Data management

Data will be recorded in paper form on CRFs by the evaluators. Under the supervision of the Ethics Committee of Nanjing University of Chinese Medicine Affiliated Hospital, all raw data will be stored at the same hospital. When recording study case notes, researchers must document the study details concurrently with patient diagnosis and treatment, ensuring timely, complete, accurate, and truthful data recording. Electronic CRFs will be used for data reporting, with designated personnel responsible for “electronic CRF data entry”. The “electronic CRF data entry personnel” will first check the study case records for completeness before entering data into the electronic CRFs. Researchers will collect data and establish a database, summarizing and analyzing the study records to generate final conclusions and reports. Upon study completion, for safety purposes, the database will be locked. Participants’ personal information will be kept confidential, and only approved researchers will have access to the data. No information will be shared with third parties without explicit written consent from the participants.

## 3 Discussion

This study is a single-center, double-blind, randomized controlled trial aimed at evaluating the efficacy of AA in improving sleep and observing its impact on the occurrence of depression following insomnia in adolescents. The goal is to provide new insights into the prevention of adolescent depression.

Insomnia is closely related to depression (34). Adolescence is a critical period for the development of personality and traits, during which adolescents also experience a “perfect storm” of sleep changes. Many factors contribute to the increased susceptibility to insomnia during this period. The reduced sleep drive in adolescence, the extended intrinsic circadian cycle of the endogenous clock, and the delayed secretion of melatonin interact with external factors such as participation in extracurricular activities, heavy homework, use of electronic devices at night, and early school start times, leading to delayed sleep onset and reduced sleep duration. Over time, these factors result in the occurrence of insomnia (35, 36). In China, the education system differs from that of other countries, with heavy homework loads and dense curricula, making academic pressure an inevitable source of stress for Chinese adolescents (37), which is one of the key factors contributing to insomnia (38).

Insomnia can trigger and maintain many negative emotions across physiological, psychological, and social domains, playing a significant role in the development of adolescent depression (39). Physiologically, insomnia increases negative emotions, reduces positive emotions, and alters adolescents’ ability to understand, express, and regulate emotions (40). Neuroimaging studies have shown dysfunction in the cortical-limbic circuits, including the prefrontal cortex (PFC), amygdala, striatum, and anterior cingulate cortex (ACC), which impair emotional responses and regulation (41–43). Additionally, insomnia causes sustained wakefulness and chronic activation of the hypothalamic-pituitary-adrenal (HPA) axis, which plays a crucial role in the development of depression (44). Psychologically, poor cognitive patterns such as worry, rumination, intrusive thoughts, and attentional biases are common in adolescents with sleep problems, and these patterns are linked to depression (45, 46). From a social perspective, insomnia reduces the likelihood of adolescents experiencing positive social environments, disrupts emotional and behavioral regulation, and harms interpersonal relationships, thereby increasing the risk of depression (40, 47).

AA, a distinctive and non-invasive modality in traditional Chinese medicine, offers operational simplicity, cost-effectiveness, and sustained therapeutic stimulation. The auricle represents the sole somatic region with cutaneous vagus nerve distribution, where key insomnia treatment acupoints (e.g., Shenmen, Heart, Liver, Kidney) coincide with dense vagal auricular branches. Stimulation of these acupoints generates neural signals that project via vagal afferents to the nucleus tractus solitarius and dorsal vagal nucleus, modulating autonomic nervous system excitability to promote sleep. Concurrently, mechanical pressure activates auricular baroreceptors, regulating somatic feedback systems to balance cerebral excitation-inhibition dynamics, thereby achieving sedative and mood-stabilizing effects. These signals further propagate to emotion-regulating brain regions (e.g., hippocampus, prefrontal cortex). Clinical evidence confirms its efficacy in improving sleep quality, emotional states, and related comorbidities (48).

However, two critical research gaps persist: only two registered auricular acupressure trials target adolescents (ITMCTR2024000343, ITMCTR2100005080), while prospective preventive studies remain absent. This school-based intervention demonstrates feasibility through standardized protocols requiring minimal training, brief 5–7 minute sessions during breaks, and low-cost materials. The combined professional administration and parent-supervised home application ensures adherence without disrupting academics, offering a replicable model for educational settings.

This study used the Chinese version of the PSQI to evaluate adolescents’ sleep quality. the psycholemtric properties of the Chinese version of the PSQI have been validated in a large sample of 5,399 by Cheng (27). The scoring algorithm for the sleep duration item retained the original recommended hours for adults rather than reflecting the longer sleep needs of adolescents. Nevertheless, the findings from the confirmatory factor analysis supported a three-factor model, with configural and metric invariance across gender and age groups, indicating that the Chinese version of the PSQI is a valid and reliable measure of sleep quality in non-clinical adolescent populations.

This study also has some limitations. First, the study’s generalizability may be limited by its exclusive focus on secondary school students in Nanjing due to budgetary and staffing constraints, which could introduce selection bias. This may introduce selection bias. Second, this is a single-center design, and single-center trials often involve a homogeneous sample, limiting generalizability and potentially introducing experimental bias. Future studies should consider more influencing factors and further validate and explore the results in larger sample populations. Third, the study outcomes rely on self-report questionnaires, which, although widely used, may still be subject to subjective biases.
